# Impact of Vein Wall Hyperelasticity and Blood Flow Turbulence on Hemodynamic Parameters in the Inferior Vena Cava with a Filter

**DOI:** 10.3390/mi16010051

**Published:** 2024-12-31

**Authors:** Jafar Moradicheghamahi, Debkalpa Goswami

**Affiliations:** 1 Liryc-Electrophysiology and Heart Modeling Institute, Fondation Bordeaux Université, 33604 Pessac, France; jafar.moradicheghamahi@ihu-liryc.fr; 2 Institute of Mathematics of Bordeaux, University of Bordeaux, 33400 Talence, France; 3 Department of Cardiovascular Medicine, Heart, Vascular & Thoracic Institute, Cleveland Clinic, Cleveland, OH 44195, USA; 4 School of Medicine, Case Western Reserve University, Cleveland, OH 44106, USA

**Keywords:** inferior vena cava filter, hyperelastic and rigid wall models, turbulent and laminar flow, hemodynamic parameters, fluid structure interaction

## Abstract

Inferior vena cava (IVC) filters are vital in preventing pulmonary embolism (PE) by trapping large blood clots, especially in patients unsuitable for anticoagulation. In this study, the accuracy of two common simplifying assumptions in numerical studies of IVC filters—the rigid wall assumption and the laminar flow model—is examined, contrasting them with more realistic hyperelastic wall and turbulent flow models. Using fluid–structure interaction (FSI) and computational fluid dynamics (CFD) techniques, the investigation focuses on three hemodynamic parameters: time-averaged wall shear stress (TAWSS), oscillatory shear index (OSI), and relative residence time (RRT). Simulations are conducted with varying sizes of clots captured in the filter. The findings show that, in regions of high wall shear stress, the rigid wall model predicted higher TAWSS values, suggesting an increased disease risk compared to the hyperelastic model. However, the laminar and turbulent flow models did not show significant differences in TAWSS predictions. Conversely, in areas of low wall shear stress, the rigid wall model indicated lower OSI and RRT, hinting at a reduced risk compared to the hyperelastic model, with this discrepancy being more evident with larger clots. While the predictions for OSI and TAWSS were closely aligned for both laminar and turbulent flows, divergences in RRT predictions became apparent, especially in scenarios with very large clots.

## 1. Introduction

Pulmonary embolism (PE), a life-threatening medical emergency, occurs when one or more pulmonary arteries are abruptly blocked, usually by blood clots that have traveled from other parts of the body, predominantly from the deep veins in the legs [1]. This blockage significantly disrupts blood flow to the lungs, leading to lowered oxygen levels and placing considerable strain on the heart [2]. The clinical symptoms of PE range from mild issues like dyspnea and chest pain to severe conditions such as respiratory failure and circulatory collapse, which can potentially lead to death [3]. The main precursor to PE is deep vein thrombosis (DVT), which involves the formation of blood clots in the deep veins, often in the lower extremities [4].

To prevent the migration of clots to the lungs and reduce the risk of PE, inferior vena cava (IVC) filters are often placed, especially in patients who cannot use or do not respond to anticoagulation therapy. These metallic devices are implanted in the largest vein of the body, the IVC, where they trap clots and prevent them from reaching the heart and lungs [5,6].

However, assessing the effectiveness of IVC filters can be challenging when relying solely on clinical reports [7]. The efficacy and benefits of these filters have been a topic of considerable debate. While some clinical studies highlight the positive impact of IVC filters on patients [8], other research suggests that their implantation may be ineffective, especially in patients already undergoing anticoagulant therapy [9].

The complexity of hemodynamics around IVC filters, combined with the varied anatomical and pathological conditions in patients, presents significant challenges for in vivo and experimental studies, often leading to inconclusive results [6]. Numerical simulations emerge as a powerful alternative, enabling the exploration of different filter designs, patient-specific anatomies, and clot scenarios in a controlled, replicable setting. These simulations utilize computational fluid dynamics (CFD) and finite element analysis (FEA) to predict flow patterns, wall stresses, and the efficiency of filters in trapping clots [10,11]. Additionally, they allow for the evaluation of hypothetical scenarios, which can inform and refine design choices without exposing patients to unnecessary risks [12]. This non-invasive, thorough, and customizable approach positions numerical simulations as an essential tool in enhancing our understanding of IVC filter behavior and the filters’ implications in clinical practice.

In biomechanical simulations, particularly those modeling blood flow in the IVC, simplifying assumptions are often necessary. The low velocity and pressure in the IVC typically justify the use of a laminar flow assumption [13,14]. However, when incorporating an IVC filter, especially one with captured clots, the validity of this assumption comes into question. The presence of large clots in an IVC filter, which can nearly block the vein, alters blood flow patterns. This alteration can induce flow separation, increase shear stress, and create downstream turbulence, all linked to a higher risk of thrombosis [15,16]. Transition zones, characterized by changes in flow patterns, have been observed to occur at least one diameter downstream from these significant clots [17]. To address these complexities, this study compares laminar and turbulent flow models, highlighting the limitations of assuming laminar flow in scenarios with significant local disturbances. Simulations incorporating realistic geometries with pulsatile boundary conditions underscore the importance of accounting for bifurcated flows and local and temporal variations, which can lead to increased turbulence and necessitate more nuanced modeling approaches [18].

The use of a rigid wall assumption for the IVC wall is another common simplification in numerical studies investigating the effects of IVC filters. This assumption is often accepted within the research community due to the relatively low pressures in the IVC, which typically do not exceed 1000 kPa [10], especially when compared to the pressures in arteries like the aorta, where pressures can surpass 11,000 kPa [19]. Additionally, the minor pressure variations in the IVC support the validity of this assumption [10,20]. However, the appropriateness of this assumption might be questioned in studies that examine the effects of an IVC filter and an entrapped clot, where alterations in flow could influence the outcomes [21,22]. Introducing a filter into the IVC increases the complexity of simulations, making it more challenging to create realistic models that account for fluid–structure interactions (FSIs). This increased complexity often justifies the continued use of the rigid wall assumption. As per the current literature, there have been no studies that incorporate two-way FSIs to examine the hemodynamics of the IVC with an implanted filter. As a result, the exact impact of IVC wall elasticity on the hemodynamics around a filter containing a clot remains unexplored.

Expanding upon our previous study [10], which was about the investigation of IVC filter and clot size on hemodynamic parameters, the present study specifically aims to examine the accuracy of laminar flow and rigid wall assumptions in simulating blood flow within a realistically modeled IVC with an integrated filter. By simulating turbulent flow and hyperelastic wall conditions across a spectrum of clot sizes, this research evaluates their impact on key hemodynamic metrics: time-averaged wall shear stress (TAWSS), oscillatory shear index (OSI), and relative residence time (RRT). The objective is to ascertain the extent to which different modeling assumptions influence the behavior of these critical parameters, thereby contributing to a more nuanced understanding of IVC hemodynamics post-filter implantation. The results show that the rigid wall assumption, compared to the hyperelastic model, is suitable only for an empty filter or small clots. Likewise, the laminar flow assumption is generally accurate but less precise with very large clots.

## 2. Methods

### 2.1. Governing Equations

The incompressible unsteady Reynolds-averaged Navier–Stokes (RANS) equations, which determine fluid motion, are represented in the following general form [11,23,24]:

(1) $$\frac{\partial \rho }{\partial t}+\frac{\partial }{{\partial x}_{i}}\left(\rho u_{i}\right)=0$$

(2) $$\frac{\partial }{\partial t}\left(\rho u_{i}\right)+\frac{\partial }{{\partial x}_{j}}\left({\rho u_{i}u}_{j}\right)=-\frac{\partial p}{{\partial x}_{i}}+\frac{\partial }{{\partial x}_{j}}\left[\mu \left(\frac{\partial u_{i}}{{\partial x}_{j}}+\frac{\partial u_{j}}{{\partial x}_{i}}-\frac{2}{3}\delta_{ij}\frac{\partial u_{k}}{{\partial x}_{k}}\right)\right]+\frac{\partial }{{\partial x}_{j}}\left(-\rho \bar{\overset{´}{u_{i}}{\overset{´}{u}}_{j}}\right)+F_{i}$$

The variable ρ denotes the density of the fluid, indicating its mass per unit volume. The temporal aspect of the flow properties is represented by the time variable t. The velocity components $u_{i}$, $u_{j}$, and $u_{k}$ correspond to the velocities of the fluid in different spatial directions. Spatial coordinates $x_{i}$, $x_{j}$, and $x_{k}$ specify the location in the fluid for evaluating variables such as velocity, pressure, and density. The pressure within the fluid is indicated by p, while μ represents the dynamic viscosity, expressed in Pascal-seconds (Pa ⋅ s), reflecting its resistance to deformation under shear or tensile stresses. Here, $\delta_{ij}$ is the Kronecker delta, defined as $\delta_{ij}=1$ when *i = j* and $\delta_{ij}=0$ when *i* ≠ *j*. The fluctuating components of velocity, $u_{i}^{\prime }$ and $u_{j}^{\prime }$, represent deviations from the mean velocity, crucial for capturing turbulence. The Reynolds stress tensor components, $\bar{u_{i}^{\prime }u_{j}^{\prime }}$, are the averaged products of these fluctuations, depicting momentum transport due to turbulence. Finally, $F_{i}$ includes external forces per unit mass acting on the fluid, which vary depending on the specific application.

The Carreau model was employed to represent the shear-thinning behavior of blood viscosity:

(3) $$\mu (\dot{\gamma })=\mu_{\infty }+(\mu_{0}-\mu_{\infty }{)[1+{\left(\lambda \dot{\gamma }\right)}^{2}]}^{(n-1)/2}$$

In this equation, μ is the apparent viscosity, $\mu_{0}=0.056\;Pa.s$ is the zero-shear-rate viscosity, $\mu_{\infty }=0.0035\;Pa\cdot s$ is the infinite-shear-rate viscosity, *n =* 0.3216 is the power-law index, *λ* = 3.313 s is the time constant, and $\dot{\gamma }$ is the shear rate. These parameter values were adopted from Cho and Kensey [25], who fitted the Carreau model to experimental data from prior studies. While not experimentally derived in their work, these parameters are widely regarded as reliable and commonly used in computational hemodynamics research.

The term $\frac{\partial }{{\partial x}_{j}}(-\rho \bar{\overset{´}{u_{i}}{\overset{´}{u}}_{j}})$ in Equation (2) is omitted for laminar flows. This term is modeled as follows for turbulent flows:

(4) $$\mu_{t}S^{2}=-\rho \bar{\overset{´}{u_{i}}\overset{´}{u_{j}}}\frac{\partial u_{j}}{\partial x_{i}}\;\;\;\;\;\;with\;\;\;\;\;\;S=\sqrt{2S_{ij}S_{ij}}$$

where *S* represents modulus of the mean strain rate tensor, and $\mu_{t}$ is the turbulence viscosity of the blood. The value of $\mu_{t}$ in the *k*-*ω* turbulence model used in this study is determined as:

(5) $$\mu_{t}=\alpha^{*}\frac{\rho k}{\omega }$$

where $\alpha^{*}$ is a correction factor for low Reynolds numbers that damps the turbulent viscosity, ρ is the fluid density, k is the turbulent kinetic energy, and ω is the rate of dissipation [23]. Equations (6) and (7) calculate the values of *k* and *ω*, respectively:

(6) $$\frac{\partial }{\partial t}\left(\rho k\right)+\frac{\partial }{{\partial x}_{i}}\left({\rho ku}_{i}\right)=\frac{\partial }{{\partial x}_{j}}\left[(\mu +\frac{\mu_{t}}{\sigma_{k}})\frac{\partial k}{{\partial x}_{j}}\right]+G_{k}-Y_{k}+S_{k}$$

(7) $$\frac{\partial }{\partial t}\left(\rho \omega \right)+\frac{\partial }{{\partial x}_{i}}\left({\rho \omega u}_{i}\right)=\frac{\partial }{{\partial x}_{j}}\left[(\mu +\frac{\mu_{t}}{\sigma_{\omega }})\frac{\partial \omega }{{\partial x}_{j}}\right]+G_{\omega }-Y_{\omega }+S_{\omega }$$

In these equations, $G_{k}$ represents the generation of turbulence kinetic energy due to mean velocity gradients, while $G_{\omega }$ represents the generation of *ω* under similar conditions. The dissipation of *k* and *ω* due to turbulence is represented by $Y_{k}$ and $Y_{\omega }$, respectively. Additionally, $S_{k}$ and $S_{\omega }$ are user-defined source terms [23].

Generally, the solid elastodynamics momentum equation is used to express the mechanical behavior of the artery wall [26]:

(8) $$\rho_{s}{\ddot{u}}_{s}=\nabla_{0}\cdot \sigma_{s}+\rho_{s}\;f$$

where $\rho_{s}$ is the solid volumetric mass, $\ddot{u_{s}}$ represents the acceleration vector of the solid (reflecting the displacement of the artery wall), $\sigma_{s}$ denotes the Cauchy stress tensor, and f is the vector of external forces acting on the solid. This equation effectively captures the dynamics of the response of the arterial wall to forces. The relationship between the Cauchy stress tensor $\sigma_{s}$ and the strain energy potential is expressed by [27]:

(9) $$\sigma_{s}=2\;\left(\frac{\partial W}{\partial C}\right)b$$

**Figure 1:**
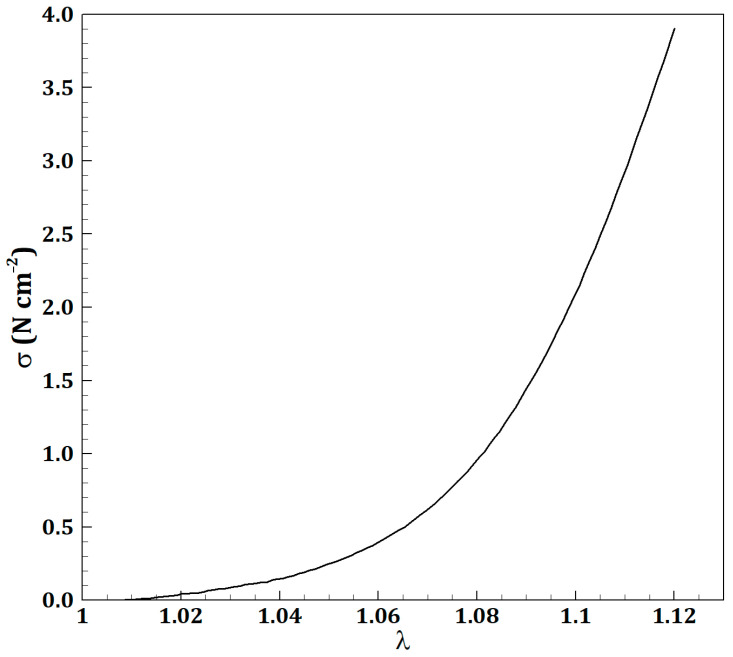
Experimental data used to derive the hyperelastic model [28,29].

In this relation, W is the strain energy potential, b is the left Cauchy–Green deformation tensor, and $C=F^{T}F,$ with *F* being the deformation gradient [27]. Considering the Yeoh hyperelastic model for the strain energy function, the potential W is defined as [23]:

(10) $$W=\Sigma_{i=1}^{N}c_{10i}{\left(I_{1}-3\right)}^{i}+\Sigma_{k=1}^{N}\frac{1}{d_{k}}{\left(J-1\right)}^{2k}$$

where W is the strain–energy potential, J is the determinant of F, and N, $c_{10i}$, and $d_{k}$ are material constants. The values of the material constants $c_{10}=0.00172\;\mathrm{MPa}$, $c_{20}=0.39449\;\mathrm{Mpa}$, and $c_{30}=5.8291\;\mathrm{Mpa}$ are based on experimental studies on the tensile properties of human IVCs [28,29] and fitted to the Yeoh third-order hyperelastic model. Figure 1, as referenced, shows the experimental data used for this fitting.

### 2.2. Problem Statement

This study builds upon our previous work [10], where the computational model was validated against the study by López et al. [14], demonstrating strong agreement in wall shear stress distributions. This prior validation forms the foundation for the current investigation, ensuring the reliability of the results. In our previous study [10], the impact of clot size on hemodynamic parameters was investigated in the context of turbulent, non-Newtonian, and pulsatile blood flow within a realistic geometry. Building on this, the current study aims to evaluate the accuracy of the commonly used rigid wall and laminar flow assumptions in numerical studies of IVC filters. To achieve this, the study compared results from simulations employing the simplifying assumptions of laminar flow and a rigid wall with simulations that incorporate more complex, realistic conditions, including turbulent flow to model blood flow and a hyperelastic model for the IVC wall, providing a more comprehensive understanding of the dynamics within the IVC filter system. This comparative approach is intended to shed light on the validity and limitations of the simplifying assumptions typically used in the study of IVC filters.

**Figure 2:**
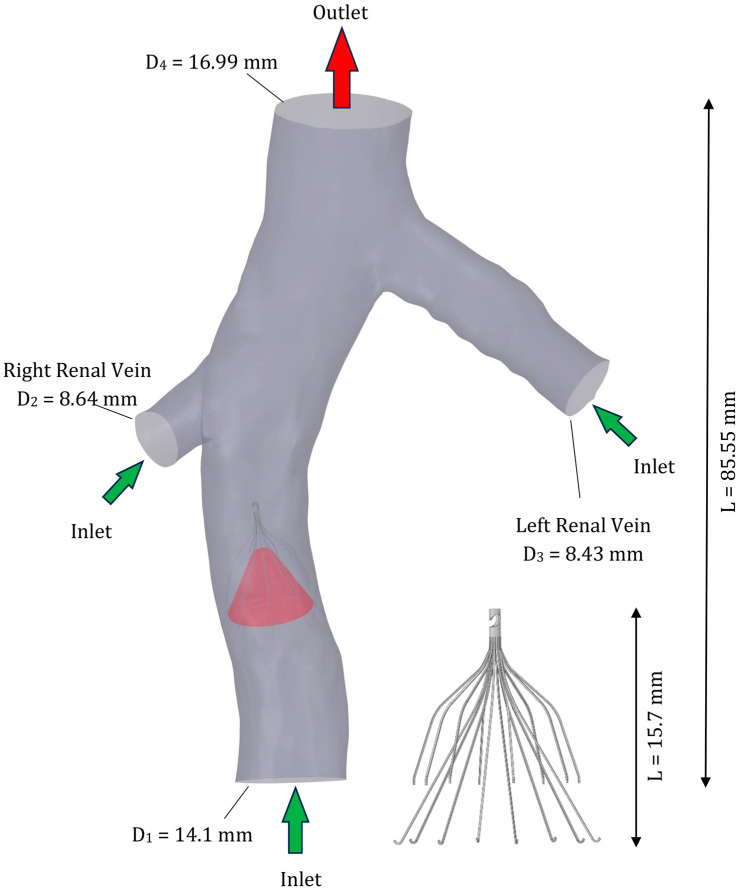
Geometry of the simulated IVC with a filter and a large clot.

In this study, the geometry of the IVC and the boundary conditions remained consistent with those used in our previous research [10]. The detailed geometry of the IVC, as shown in Figure 2, included a segment of the IVC to which the renal veins are connected. Additionally, the study utilized the Recovery filter G2 model [30], as depicted in Figure 2. The initial geometry (without a filter), sourced from CT scan data and used in previous studies [13,14], did not closely resemble an ideal cylindrical shape. To address this, the software SpaceClaim 2022 R2 (ANSYS Inc., Canonsburg, PA, USA) was employed to modify the geometry of the vein, aiming to achieve a more cylindrical configuration around the filter, as the original CT data did not capture the impact of the filter on vein shape. Studies have shown that IVC filters can exert radial force on the surrounding IVC wall, potentially altering its geometry by increasing circularity or causing localized deformation in the vein [31,32]. This modification is crucial as it enhances the contact between the filter and the vessel wall, thereby creating a more realistic simulation environment compared to previous studies. The study focused on examining the effects of three sizes of blood clots: small, medium, and large, with diameters constituting 25%, 50%, and 75% of the IVC diameter, respectively. Consistent with findings from previous studies [13,24], it is noted that the geometry of the clot tends to conform to the filter geometry. In this study, the shape of the filter resulted in a conical shape of the clot as it becomes trapped within the filter.

**Figure 3:**
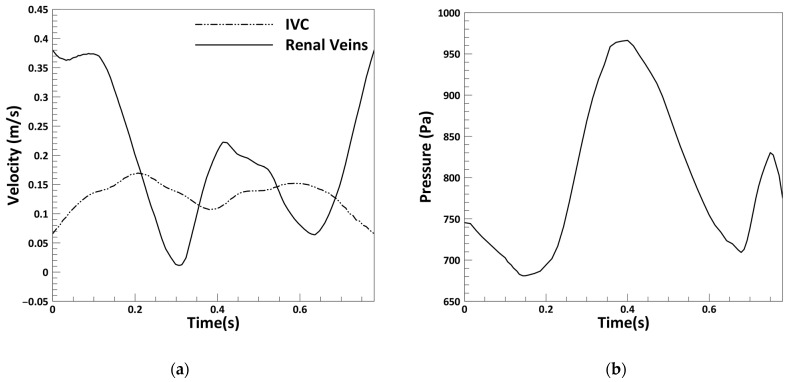
(**a**) Physiological pulses utilized at the inlet; (**b**) outlet boundary conditions [22,33].

In this study, pulsatile boundary conditions were applied to simulate blood flow in the IVC, based on physiological data from the literature (Figure 3). A velocity pulse was imposed at the inlet of the IVC, while identical velocity pulses were applied at the inlets of the left and right renal veins to represent their periodic flow patterns. At the outlet, a pulsatile pressure condition was specified. These boundary conditions were adopted from established studies [22,33], and a no-slip boundary condition was applied to the vessel walls. The study models the clot within the blood vessel as a fixed, immobile internal rigid wall. This modeling approach aligns with previous computational studies focused on IVC filters [13,14,29].

Opting not to employ a multiphase simulation, the study assumed that both the position and size of the clot remain constant throughout the simulations. This decision allows for a concentrated analysis on the impact of the clot and filter on blood flow patterns, without the added complexities of clot movement or deformation. Additionally, no-slip boundary conditions were applied to all walls in the simulations. This means that the velocity of the fluid (blood in this case) at the wall was assumed to be zero, which is a standard assumption in fluid dynamics for modeling the interaction between a fluid and a solid boundary.

**Figure 4:**
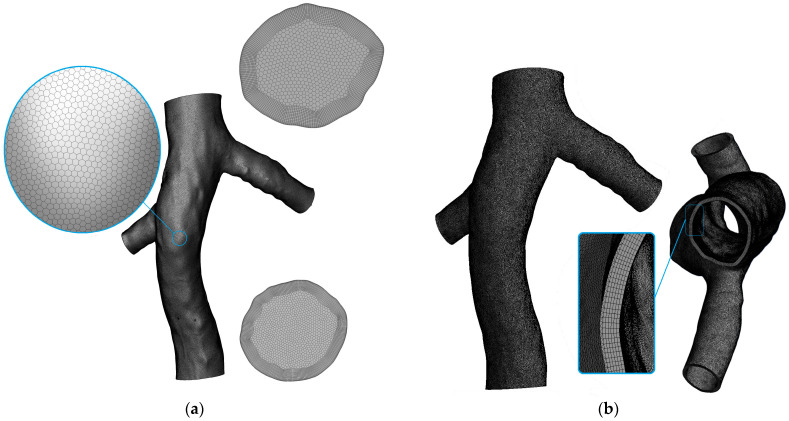
Details of the selected mesh. (**a**) Fluid domain; (**b**) Solid domain.

In this research, CFD and FSI techniques were employed. Building upon the mesh study conducted in our previous study [10], specific mesh sizes were determined for the fluid domain and the surfaces of the filter and clot. A poly-hexcore mesh was used for the fluid domain, which combines structured hexahedral cells in the core regions with unstructured polyhedral cells near the boundaries. This configuration enhances the computational efficiency while maintaining an accurate resolution in regions of complex geometry and flow [34]. For the fluid domain on the IVC surface, the minimum element size was set at 2 × 10^−4^ m, while on the filter and clot surfaces, a smaller minimum size of 5 × 10^−5^ m was used. In both scenarios, the maximum size of the mesh elements was capped at twice their minimum size. This mesh configuration led to the generation of a fluid domain mesh comprising a total of 5,611,283 cells, as shown in Figure 4a. For the solid domain, the mesh consisted of 1,215,095 total elements, each with a size of 3 × 10^−4^ m (Figure 4b). To validate the mesh adequacy of the solid domain, the simulation of the IVC without the filter was repeated using a finer mesh, which had an element size of 2 × 10^−4^ m and a total of 2,873,053 elements. This additional simulation confirmed that there was no significant difference in the hemodynamic parameters compared to the initial mesh, thus validating the mesh size chosen for this study.

In this study, the commercial CFD software ANSYS Fluent 2022 R2, coupled with the FEA software ANSYS Transient Structural 2022 R2, was employed for flow visualization and analysis. The discretization of the governing equations was achieved using a third-order Monotone Upstream-centered Schemes for Conservation Laws (MUSCL) differencing scheme, known for its accuracy in reducing numerical diffusion, thus capturing intricate flow dynamics effectively [35]. Pressure–velocity coupling was addressed using the COUPLED scheme, offering robustness and precision in solving the Navier–Stokes equations [23] within the complex geometrical and boundary conditions of the study. To ensure a high degree of accuracy, a stringent residual error convergence threshold of 10^−6^ was set for the momentum equations. Furthermore, the study conducted unsteady simulations over three consecutive pulses, as per the protocols established in previous studies [36], to mitigate transient effects, basing the analysis on the data from the third pulse for steadier state conditions. The simulation parameters, including a maximum of 40 iterations per time step and a time step size of 0.005 s, were carefully chosen. Sensitivity tests varying these parameters (time step to 0.002 s and maximum iterations to 80) showed no meaningful changes, affirming the stability and appropriateness of the selected simulation settings.

### 2.3. Hemodynamic Parameters

To assess the accuracy of the laminar model and rigid wall model against the turbulent and hyperelastic model, this study employed three wall shear stress (WSS)-based hemodynamic parameters commonly used in the study of thrombus growth and atherosclerosis [37]. These parameters are Time-Averaged Wall Shear Stress (TAWSS), Oscillatory Shear Index (OSI), and Relative Residence Time (RRT).

To quantify the average shear stress exerted on the wall during the entire cardiac cycle, TAWSS is calculated using the following equation, which has been utilized in previous studies [38,39].

(11) $$TAWSS=\frac{1}{T}\int_{0}^{T}\left|\tau_{s}\right|dt$$

The OSI is employed to evaluate the cyclic variations of shear stress that endothelial cells are continuously subjected to during a cardiac cycle. It is a useful metric for assessing the average time of flow separation and reattachment [40]. Higher OSI values indicate areas of more frequent flow direction changes. OSI varies from zero (indicating pure unidirectional flows) to 0.5 (for totally oscillatory flows) [41]. For pulsatile flows, OSI is calculated as [11,42]:

(12) $$OSI=\frac{1}{2}(1-\frac{\left|\frac{1}{T}\int_{0}^{T}\tau_{s}\;dt\right|}{\frac{1}{T}\int_{0}^{T}\left|\tau_{s}\right|\;dt})$$

The RRT assesses the distribution of frictional forces on the inner surface of the vessel and is particularly significant in regions experiencing low and oscillating wall shear stresses [43]. It measures how closely particles remain to the wall and is calculated as [11,42]:

(13) $$RRT=\frac{1}{\left(1-2\;OSI\right)\cdot TAWSS}$$

## 3. Results

### 3.1. The Effect of Hyperelasticity

#### 3.1.1. Time-Averaged Wall Shear Stress (TAWSS)

**Figure 5:**
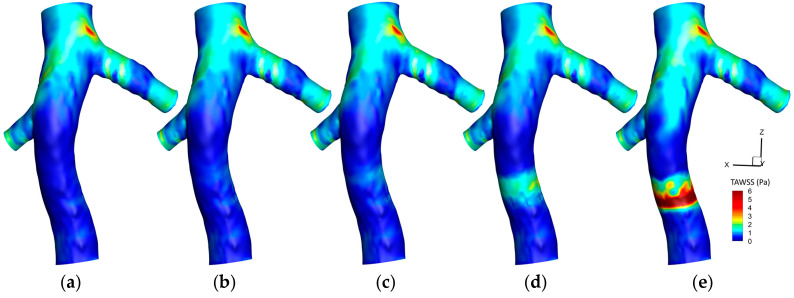
TAWSS contours on the IVC wall for different cases: (**a**) IVC without a filter; (**b**) IVC with an unoccluded filter; (**c**) IVC with a filter and a small, captured clot; (**d**) IVC with a filter and a medium captured clot; (**e**) IVC with a filter and a large, captured clot.

Figure 5 illustrates the variation in TAWSS on a hyperelastic IVC wall under different conditions. In the absence of a filter and clot, a baseline scenario is presented with a relatively consistent TAWSS distribution. Implanting a filter causes localized fluctuations in TAWSS around the filter, reflecting alterations in the flow dynamics due to the presence of the filter.

As we proceed from a small to a large clot size within the filter, there is a distinct modification in the TAWSS distribution. The regions around the filter exhibit pronounced increases in TAWSS, highlighting the enhanced hemodynamic disturbances caused by the presence of the filter and clot. Additionally, there is an observable decrease in TAWSS values downstream of the clot, particularly evident when a larger clot is present, demonstrating the significant impact of the clot size on altering the shear stress environment within the IVC. This representation underscores the multifaceted hemodynamic consequences induced by the integration of a filter and the incorporation of clots of varying magnitudes.

**Figure 6:**
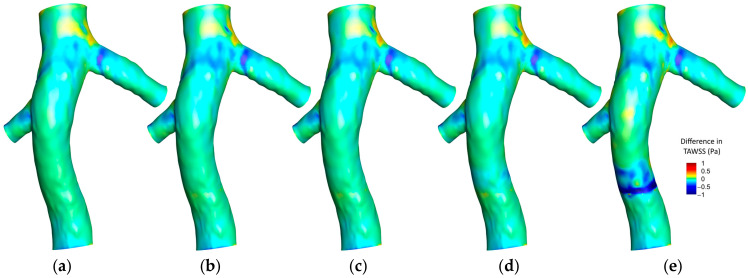
Difference between the hyperelastic and rigid wall models in TAWSS prediction on the IVC wall for different cases: (**a**) IVC without a filter; (**b**) IVC with an unoccluded filter; (**c**) IVC with a filter and a small, captured clot; (**d**) IVC with a filter and a medium captured clot; (**e**) IVC with a filter and a large, captured clot.

Figure 6 delineates the difference in the TAWSS yielded by hyperelastic and rigid wall models (TAWSS_Hyperelastic_—TAWSS_Rigid_) across various scenarios in the IVC. In various scenarios across extensive regions of the IVC wall, it is evident that there is no discernible difference between the hyperelastic and rigid wall models. In the absence of a filter, a slight difference is discernible at the location of the left renal vein conjunction, with the rigid wall model forecasting slightly elevated TAWSS values. The incorporation of a filter, without a clot, does not introduce further discrepancies between the two models. The presence of a small or medium clot also remains consistent with this observation, maintaining a minimal variance in the TAWSS predictions of the two models. However, a deviation is observed in the scenario featuring a large clot. In this instance, the rigid wall model exhibits higher TAWSS values specifically at the location of the clot, underscoring a notable sensitivity to the wall elasticity assumptions in the presence of substantial intravascular obstructions.

**Figure 7:**
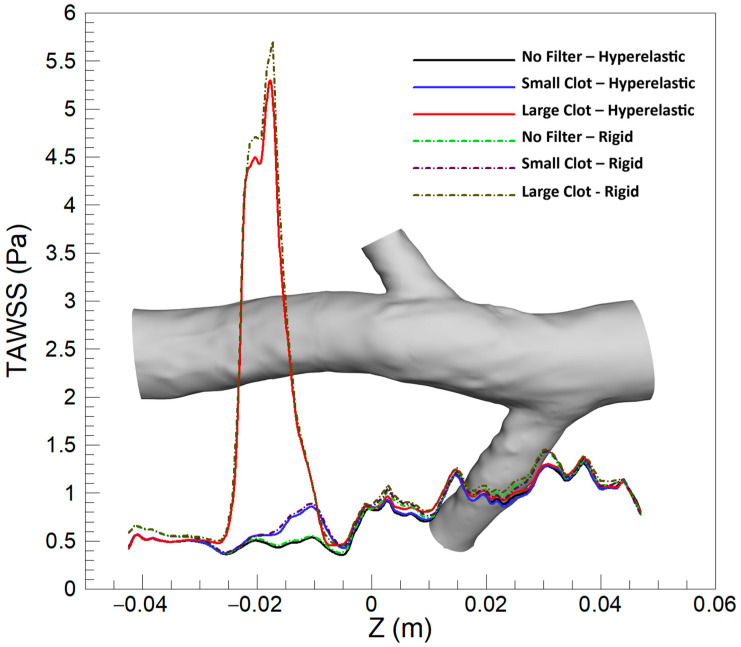
Average TAWSS along the Z-direction for three scenarios, comparing the hyperelastic and rigid wall models.

Figure 7 shows the variations in the averaged TAWSS along the Z-axis under diverse scenarios. A close observation reveals nuanced differences attributable to the wall model utilized. In the absence of a filter, there is a slight elevation in the averaged TAWSS at the conjunction of the left renal vein in the rigid wall model. For the case of the IVC with a filter containing a small clot, slight variations are observable around the filter location. The distinctions become more prominent with a larger clot, where the rigid wall model demonstrates an increase in TAWSS values around the location of the clot, emphasizing the variability in hemodynamic responses based on the wall elasticity assumptions employed in the modeling.

#### 3.1.2. Oscillating Shear Index (OSI)

**Figure 8:**
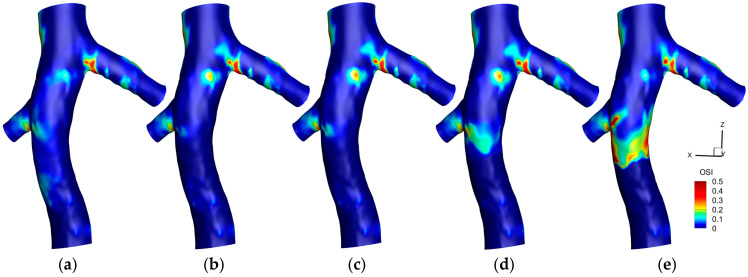
OSI contours on the IVC wall for different cases: (**a**): IVC without a filter; (**b**): IVC with an unoccluded filter; (**c**): IVC with a filter and a small, captured clot; (**d**): IVC with a filter and a medium captured clot; (**e**): IVC with a filter and a large, captured clot.

Figure 8 illustrates the distribution of the OSI on the hyperelastic IVC for various scenarios. In the absence of a filter, a specific region in the left renal vein conjunction exhibits noticeable OSI values. With the introduction of a filter, two prominent observations are made: a reduction in OSI values at the location of the filter and an increase in the region between the two renal vein conjunctions.

Adding a small clot to the filter reveals a subtle effect on the OSI distribution, marked by a slight increase just downstream of the clot, proximal to the right renal vein conjunction. This trend amplifies with a medium-sized clot, where a more noticeable elevation in OSI values downstream of the clot is observed. A significant increase is witnessed with a large clot, characterized by a broad area exhibiting heightened OSI values downstream of the clot, underlining the pronounced influence of a larger clot size on the oscillatory nature of shear stresses within the IVC.

**Figure 9:**
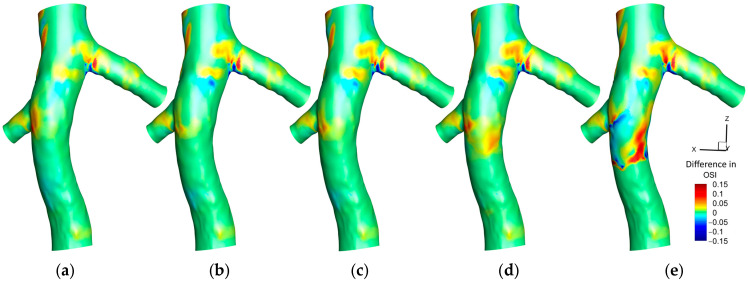
Difference between the hyperelastic and rigid wall models in OSI prediction on the IVC wall for different cases: (**a**): IVC without a filter; (**b**): IVC with an unoccluded filter; (**c**): IVC with a filter and a small, captured clot; (**d**): IVC with a filter and a medium captured clot; (**e**): IVC with a filter and a large, captured clot.

Figure 9 illustrates the difference in OSI outcomes between the hyperelastic and rigid wall models (OSI_Hyperelastic_—OSI_Rigid_) on the IVC wall under different conditions. In scenarios without a filter, with an empty filter, and with a filter containing a small clot, a pronounced difference is noticeable at the left renal vein conjunction, where the hyperelastic model predicts a higher OSI. As the clot size enlarges, the disparity between the two models accentuates just downstream of the clot, with the hyperelastic model predicting higher OSI values. This divergence broadens with the increase in the clot size, culminating in a distinct, wide area of notable difference downstream of a large clot.

**Figure 10:**
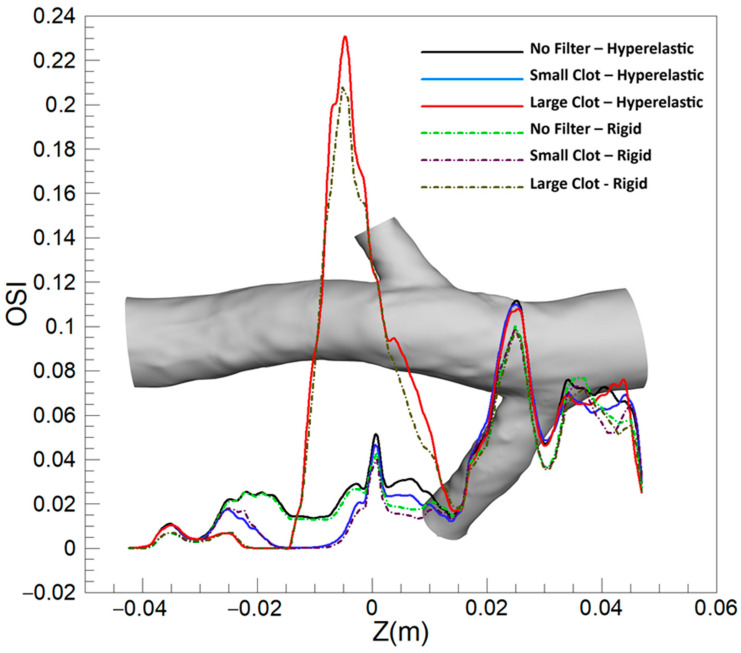
Average OSI along the Z-direction for three scenarios, comparing the hyperelastic and rigid wall models.

Figure 10 illustrates the average OSI along the Z-direction in the IVC, covering three different scenarios: without a filter, with a small clot, and with a large clot. Each scenario is analyzed using both the hyperelastic and rigid wall models, revealing that the discrepancies in average OSI between the two models are generally more pronounced than those observed in TAWSS. Notably, a significant difference is evident at the location of the left renal vein conjunction across all scenarios. A meaningful difference is also present at the conjunction of the right renal vein. The scenario involving a large clot exhibits the most considerable disparity between the two models, particularly noticeable downstream of the clot, emphasizing the substantial impact of wall elasticity assumptions in the presence of larger intravascular obstructions.

#### 3.1.3. Relative Residence Time (RRT)

**Figure 11:**
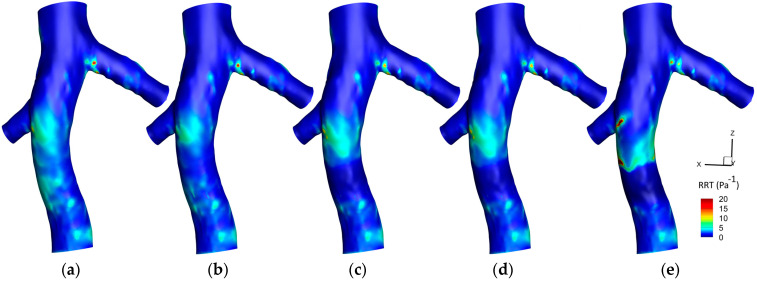
RRT contours on IVC wall for different cases: (**a**): IVC without a filter; (**b**): IVC with an unoccluded filter; (**c**) IVC with a filter and a small, captured clot; (**d**) IVC with a filter and a medium captured clot; € IVC with a filter and a large, captured clot.

Figure 11 displays the RRT contours on the hyperelastic IVC wall across five different scenarios. Initially, without the filter, an area of high RRT is discernible at the conjunction of the left renal vein. Moderate values of RRT are also observed preceding the conjunction of the right renal vein. The implementation of a filter subtly reduces the intensity of RRT around the filter location. This pattern is slightly augmented when a small clot is captured by the filter, coupled with a subtle increase in RRT values downstream of the clot. A medium-sized clot accentuates these variations further, showcasing a more explicit reduction in RRT proximally to the filter and a pronounced increase downstream of the clot. In the scenario involving a large clot, a distinctive region surrounding the clot exhibits minimized RRT values, contrasted by elevated levels of RRT downstream of the clot. This is also accompanied by a more substantial increase in RRT at the conjunction of the right renal vein, underscoring the significant influence of clot size in modulating the flow residence times and hemodynamic behavior within the IVC.

**Figure 12:**
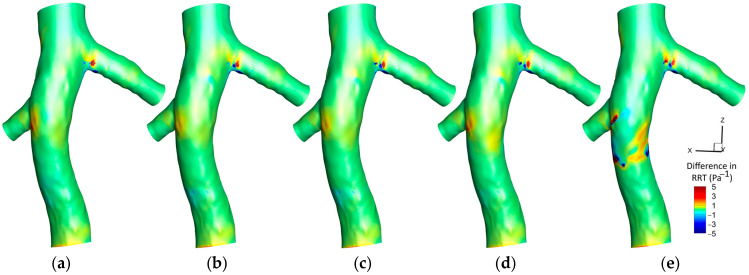
Difference between the hyperelastic and rigid wall models in RRT prediction on IVC wall for different cases: (**a**): IVC without a filter; (**b**) IVC with an unoccluded filter; (**c**) IVC with a filter and a small, captured clot; (**d**) IVC with a filter and a medium captured clot; (**e**) IVC with a filter and a large, captured clot.

Figure 12 illustrates the difference between the RRT contours predicted by the hyperelastic and rigid wall (RRT_Hyperelastic_—RRT_Rigid_) models on IVC wall, across various scenarios. Initially, prior to filter implantation, elevated RRT values are discerned at the conjunction of the left renal vein in the hyperelastic model. Additionally, a distinct region preceding the conjunction of the right renal vein demonstrates higher RRT values in the hyperelastic model, a distinction that intriguingly diminishes upon filter implantation and reemerges with the increase in the clot size. Except in the scenario involving a large clot, there is a minimal discrepancy in the RRT downstream of the clot between the two models. However, the presence of a large clot unveils a significant difference, with the hyperelastic model indicating higher RRT values. The figure predominantly showcases regions where the hyperelastic model estimates increased the RRT compared to the rigid wall model.

**Figure 13:**
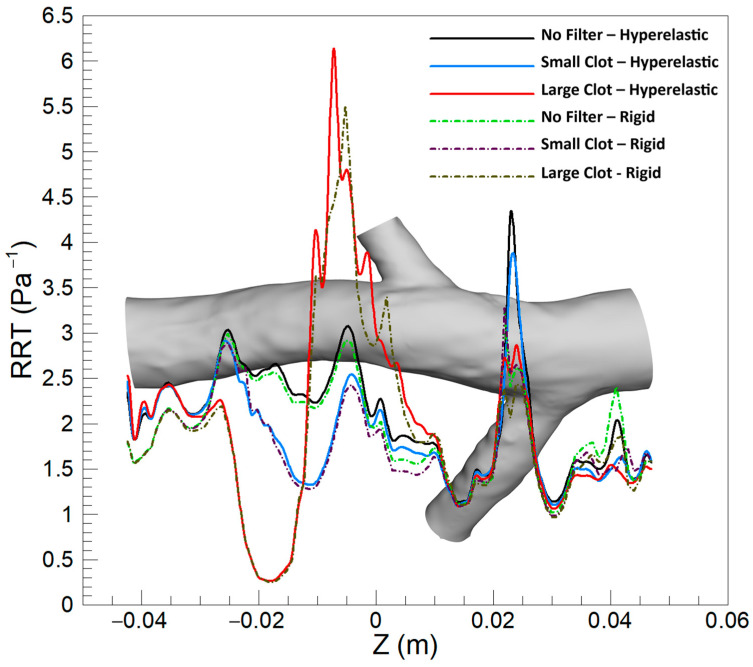
Average RRT along the Z-direction for three scenarios, comparing the hyperelastic and rigid wall models.

Figure 13 exhibits the average RRT along the Z-direction across different scenarios, comparing the hyperelastic and rigid wall models. Overall, the hyperelastic model tends to predict slightly elevated average RRT values compared to the rigid wall model. In scenarios without a filter and that involving a small clot, the most substantial difference between the two models manifests at the conjunction of the left renal vein. Conversely, in the case of a large clot, the maximal discrepancy in predictions is observed downstream of the clot. A notable observation is the variance in the location of the maximum average RRT as predicted by each model in the presence of a large clot, indicating a shift in the regions identified as having the highest residence times based on the wall model applied.

### 3.2. The Effect of Turbulence

The influence of turbulence on hemodynamic parameters is less significant compared to the impact of hyperelasticity. Given that the high degree of similarity observed in the 3D contours of hemodynamic parameters resulted in the simulations using turbulent or laminar blood flow, only the 2D graphs representing the average parameters along the Z-direction are presented in this section.

#### 3.2.1. TAWSS

**Figure 14:**
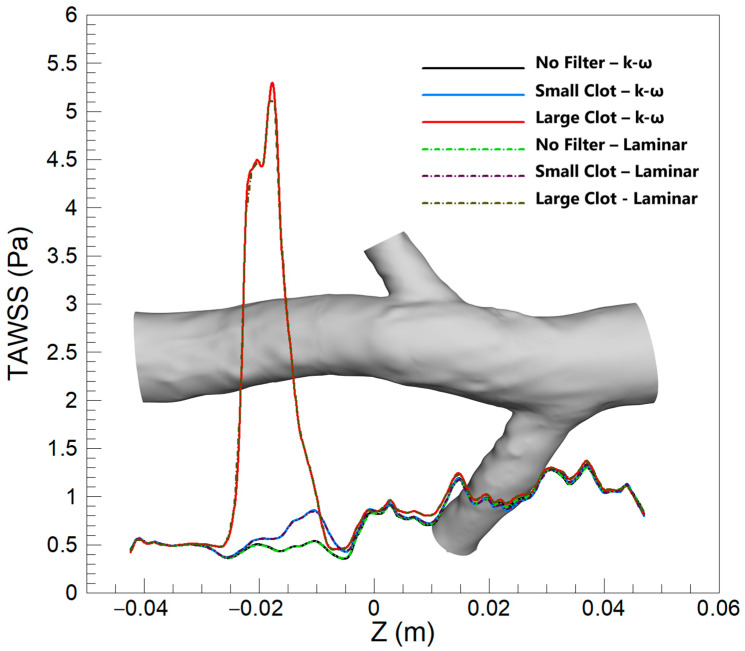
Average TAWSS along the Z-direction for three scenarios, comparing the k-ω and laminar models.

Figure 14 illustrates the average TAWSS along the Z-direction for various scenarios, and for both turbulent and laminar blood flow models. The figure reveals that there is no substantial difference in TAWSS predictions between the two models overall. However, a slight divergence is observed in the case of a large clot at the location of the clot, where the turbulent model predicts a slightly elevated average TAWSS value compared to the laminar model. This distinction, although present, is relatively minor when compared to the differences observed between the hyperelastic and rigid wall models in the previous sections.

#### 3.2.2. OSI

**Figure 15:**
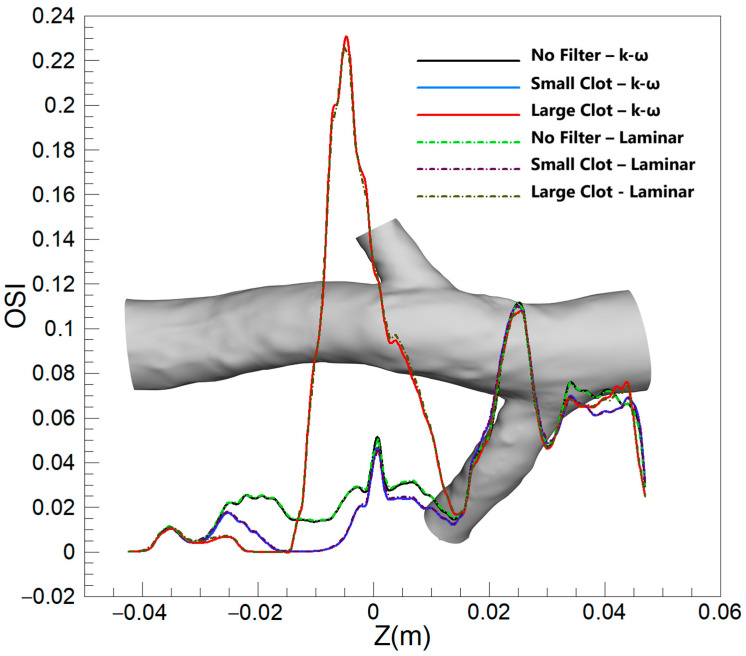
Average OSI along the Z-direction for three scenarios, comparing the k-ω and laminar models.

Figure 15 displays the averaged OSI along the Z-direction under various scenarios, comparing outcomes between turbulent and laminar blood flow models. The depiction reveals a broad similarity between the two models in predicting OSI values across different conditions, echoing observations made for the TAWSS parameter. The models yield almost identical predictions for various scenarios and locations, demonstrating a consensus in capturing the oscillatory behavior of flow within the vessel. A minor discrepancy emerges downstream of the clot in scenarios involving a larger clot size, where the turbulent model slightly overestimates the average OSI compared to the laminar model.

#### 3.2.3. RRT

**Figure 16:**
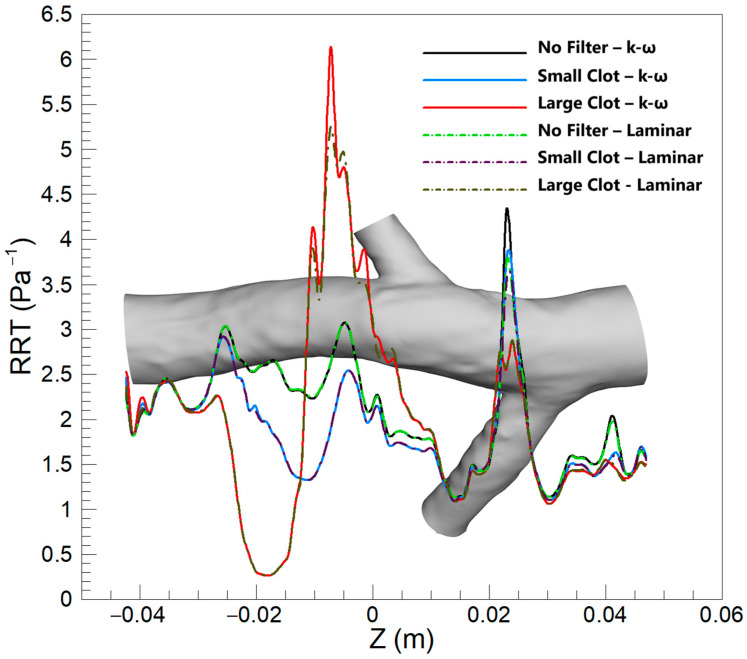
Average RRT along the Z-direction for three scenarios, comparing the k-ω and laminar models.

Figure 16 exhibits the average RRT along the Z-direction for a variety of scenarios, for both the turbulent and laminar blood flow models. In contrast to the observations made for TAWSS and OSI, noticeable differences in RRT predictions emerge between the two flow models, in scenarios without a filter and with a small clot. In these scenarios, at the conjunction of the left renal vein, the turbulent model predicts a marginally higher average RRT compared to the laminar model. Nevertheless, these disparities are modest when contrasted with the differences observed between the hyperelastic and rigid wall models. Furthermore, the most significant difference in results between the two flow models occurs downstream of the clot in the scenario featuring a large clot, where the turbulent model forecasts a higher average RRT. Despite this, the magnitude of this difference is still relatively subdued when compared to the variations between the hyperelastic and rigid wall models.

### 3.3. Mean Values in Critical Areas

At the critical areas identified in Figure 7, Figure 10 and Figure 13, the mean values of TAWSS, OSI, and RRT were calculated and are presented in Table 1. For TAWSS, the critical area spans a *z*-coordinate range from −0.025 m to −0.01 m, while for OSI and RRT, the critical area spans from −0.015 m to 0.015 m. For the small clot case, the rigid wall model and laminar flow assumption yielded results that closely matched those of the hyperelastic wall and turbulent models, with percentage differences below 3.1% for mean TAWSS, 1.3% for mean OSI, and 2.5% for mean RRT. In contrast, for the large clot case, the percentage differences increased substantially, with deviations reaching 6.05% for mean TAWSS, 4.4% for mean OSI, and 12.06% for mean RRT when comparing the rigid and hyperelastic wall models. The differences between the turbulent and laminar flow models were generally smaller, with a maximum difference of 1.2% for mean TAWSS, 3.2% for mean OSI, and 10.34% for mean RRT in the large clot scenario.

## 4. Discussion

### 4.1. TAWSS

For determining the rate of progression of cardiovascular diseases like atherosclerosis and thrombosis, a reduction in the value of TAWSS on the vessel wall is generally the most important factor [44,45]. A low wall shear stress (WSS) can lead to thrombus formation, endothelial cell dysfunction and damage, and low oxygen tension. Platelets can adhere to the endothelial cells lining the vessel wall, leading to the formation of a platelet-rich thrombus [46]. Low WSS can cause endothelial cells to produce less nitric oxide, upregulate pro-inflammatory cytokines and adhesion molecules, promoting platelet aggregation and thrombus formation [47]. Additionally, low WSS can create areas of low oxygen tension, resulting in the release of more adenosine diphosphate (ADP) from red blood cells, which is a potent activator of platelets [48].

In Figure 5, the implantation of the IVC filter is associated with a marginal decline in TAWSS, a trend that is accentuated when a large clot is present. This observation suggests an increased susceptibility to thrombosis in regions downstream of the clot. Figure 6 and Figure 7 further reveal that the TAWSS values predicted by the rigid wall model are slightly higher than those predicted by the hyperelastic model. This discrepancy is attributed to the ability of the hyperelastic model to simulate the deformation of the IVC wall, which, in the presence of a large clot, yields an expanded cross-sectional area for blood flow, thereby diminishing both the velocity and its gradient near the vessel wall. This phenomenon is particularly evident in cases with a large clot, indicative of significant wall deformation under such conditions. Despite these differences, the rigid wall model provides reasonably accurate predictions of TAWSS when compared to the more physiologically representative hyperelastic model, particularly in scenarios without extreme vessel deformation.

Although low shear stress has always been cited as a cause of vascular occlusion diseases [49,50], high shear stress can also cause these types of diseases [51,52]. High shear stresses and turbulence cause damage to endothelial cells [53], play an important role in platelet activation and may cause plaque rupture [54]. This is due to the creation of regions with high shear stress along the arterial wall. As a result, inflammation and further weakening of the arterial wall can occur [55]. Additionally, high WSS values can change blood flow patterns, potentially leading to aneurysm growth and rupture. For instance, vortex flows may form within the aneurysm sac, contributing to thrombus formation and further weakening of the arterial wall [56].

In Figure 5, the introduction of a filter and subsequent clot placement are shown to result in an elevation of TAWSS within their immediate vicinity. Notably, with the insertion of a medium-sized clot, the TAWSS remains within a moderate range. However, the presence of a large clot sees TAWSS values surpassing the threshold of 5 Pa, a critical value for platelet activation as identified in the existing studies [46,57]. Such high TAWSS regions are thus characterized as being susceptible to thrombotic events, particularly in instances where a large clot is entrapped by the filter. Further examination of Figure 6 and Figure 7 reveals a discernible discrepancy in TAWSS predictions between the hyperelastic and rigid wall models, with the latter consistently estimating higher TAWSS. This difference underscores the significance of wall compliance in the mechanical environment experienced by the vascular endothelium and its potential role in thrombus formation.

In scenarios where an IVC filter has captured a large clot, there is a notable obstruction to blood flow. Due to the principle of continuity, blood velocity is increased at the point of constriction to maintain flow rate, which, in turn, elevates shear forces on the vessel wall. Furthermore, the presence of the clot influences pressure gradients along the vessel, with an intensified gradient at the obstruction potentially leading to increased shear forces along the walls adjacent to the clot. In contrast, hyperelastic vessel walls, unlike rigid ones, are capable of stretching and deforming in response to variations in blood flow pressure. This adaptability to increased upstream pressure, especially in the presence of a large clot, permits the vessel wall to expand. Such expansion increases the cross-sectional area available for blood flow, which can reduce the blood velocity near the walls and thus lower the WSS. Disparities in wall shear stress predictions are observed, with differences exceeding 15% in specific regions, as demonstrated in Figure 6. Additionally, as shown in Figure 7, the average TAWSS prediction from rigid wall assumptions can be about 10% higher in these areas, which suggests that rigid wall models may overestimate the risk of thrombosis by predicting elevated levels of TAWSS.

In Figure 14, the TAWSS predictions align closely between the laminar and turbulent flow models under various scenarios. This congruence is attributed to typically low velocities of blood flow in the vein, suggesting laminar flow prevails. However, a notable clot within the IVC filter alters flow dynamics, increasing velocity and Reynolds number locally, which may shift conditions towards turbulence. This shift is reflected marginally in the predictions around the large clot, where the turbulent model, sensitive to the local flow disturbances, predicts a slightly higher TAWSS due to turbulent eddies. Despite this, the overall agreement between models indicates that laminar flow assumptions predominantly suffice in simulating the hemodynamics of the IVC, even when a filter is present. The data suggest that the laminar model maintains a high degree of accuracy in evaluating thrombosis risk, reaffirming its validity in both low and elevated shear stress regions.

The quantitative analysis reveals that, for small clots, the rigid wall and laminar flow models predict mean TAWSS values that closely align with those of the hyperelastic wall and turbulent flow models, with differences remaining below 3.1%. For large clots, however, the difference between the rigid wall and hyperelastic models increases to 6.05%, reflecting the limitations of the rigid wall assumption in accurately capturing the complex deformation of the vessel wall under significant flow disturbances. In contrast, the differences between the turbulent and laminar flow models are smaller, with a maximum difference of 1.2% observed for mean TAWSS in the large clot scenario. These findings suggest that the laminar flow assumption generally performs well in predicting mean TAWSS across various scenarios, although its accuracy may be slightly affected in cases involving significant flow disturbances and higher Reynolds numbers.

### 4.2. OSI

Oscillatory or disturbed blood flow, as indicated by OSI values, can precipitate endothelial dysfunction due to the response of the endothelial cells to variable shear stresses [58]. This dysfunction promotes a pro-inflammatory and pro-thrombotic environment, increasing thrombus formation risk as blood flow patterns are disrupted, leading to cell stagnation and the activation of coagulation pathways [59]. Vascular remodeling, including atherogenic-like changes such as neointimal hyperplasia, may also ensue from sustained oscillatory shear stress, altering the structure of the vessel wall and mechanical properties [60]. Experimental findings have corroborated that sites of thrombosis formation are characterized by high OSI and low shear stress, substantiating the relationship between local hemodynamic conditions and occlusion risk [61,62,63]. OSI has been validated against clinical data as a reliable predictor of disease risk, underscoring its relevance in vascular health assessment [64].

As depicted in Figure 8, moderate OSI values are observed upstream of the right renal vein. The introduction of an IVC filter precipitates a reduction in OSI within this region, likely due to the influence of the filter on hemodynamics, streamlining flow and promoting more uniform, unidirectional patterns that diminish the oscillatory shear stress on the vessel wall. Upon the addition of a small clot, a subtle escalation in OSI is detected, resulting from flow disturbances such as localized eddies and flow separation induced by the clot. This disturbance intensifies with an increase in clot size, with larger clots causing significant obstruction and consequently fostering flow diversion, recirculation, and vorticity downstream—effects that are amplified in the clot’s wake. In scenarios involving a large clot, these hemodynamic alterations lead to pronounced oscillatory flow downstream, significantly increasing OSI values prior to the right renal vein. The combination of low TAWSS and elevated OSI in this area indicates an increased susceptibility to thrombosis.

In Figure 9, the elevated OSI values predicted by the hyperelastic model at the left renal vein conjunction can be attributed to the enhanced capacity of the model to replicate the dynamic and elastic response of the vessel wall to pulsatile flows. The flexible nature of the hyperelastic wall results in increased motion, amplifying oscillations in the blood flow at this anatomically complex region, in contrast to the rigid wall model that fails to account for such dynamic interactions, thus predicting lower OSI values. It is noteworthy, as indicated in Figure 8, that the OSI values associated with an empty filter are comparatively lower, with both models yielding more congruent predictions in this instance. However, Figure 9 highlights that the discrepancy in OSI predictions between the two models escalates with increased flow complexity (due to addition of captured clots), particularly downstream of the filter. This gap widens progressively with clot size; the hyperelastic model consistently indicates higher OSI levels, especially downstream of the filter. This trend is evident in Figure 10, where the hyperelastic model predicts the average OSI downstream of the clot to be approximately 10% higher in the large clot scenario. This divergence in model predictions arises as larger clots disrupt blood flow more substantially, heightening oscillatory shear stress in the hyperelastic model. Conversely, the limitations of the rigid model in simulating elasticity leads to an underestimation of these stress oscillations, which is particularly evident with larger clots that introduce significant flow disturbances. Additionally, given the rigid wall’s slightly lower predictions of TAWSS in this region, it can be inferred that it underestimates the thrombotic risk downstream of the clot compared to the hyperelastic model.

Figure 15 illustrates that, across a spectrum of scenarios, the OSI predictions from laminar and turbulent flow models are remarkably similar, underscoring the predominantly laminar flow characteristics of the venous system. Nonetheless, with a large clot present, there is a marginal increase in OSI values predicted by the turbulent model downstream of the clot. This is indicative of the enhanced sensitivity of the turbulent model to localized disturbances such as eddies and instabilities, which arise from the interaction of the flow with the clot—complexities that the laminar model does not fully resolve. Nonetheless, the precision of the predictions of the laminar model remains notable when evaluated against the turbulent model, which serves as a benchmark, affirming its appropriateness for OSI assessments in IVC with filter.

A closer examination of the mean OSI values reveals that, for small to moderate clots, the predictions from the rigid wall and laminar flow models align closely with those of the hyperelastic wall and turbulent flow models, with differences remaining below 1.3% and 1.8%, respectively. However, for large clots, the difference between the rigid wall and hyperelastic models increases to 4.4%, indicating the reduced capability of the rigid wall model to capture the effects of vessel wall deformation on oscillatory flow patterns. Similarly, the turbulent flow model predicts slightly higher mean OSI values (up to 3.2%) compared to the laminar flow model, particularly in regions where flow disturbances are pronounced due to clot presence. These findings suggest that, while the laminar flow and rigid wall assumptions generally perform well for small clots, their accuracy decreases in capturing flow oscillations in scenarios with significant flow complexity and deformation caused by large clots.

### 4.3. RRT

The relationship between particle residence time, OSI, and TAWSS is intricately linked to thrombogenic processes [65]. An elevated OSI often suggests a diminished TAWSS, a combination indicative of potential vascular damage [66]. Yet, high OSI regions do not invariably overlap with areas of low TAWSS, necessitating a careful spatial analysis within vascular studies [67]. RRT is utilized as a robust metric to pinpoint regions characterized by both low shear stress and high oscillatory flow [43]. Such zones are of particular interest due to their association with stagnated blood flow, which heightens the thrombosis risk by prolonging endothelial contact with blood [46]. This stagnation may compromise the endothelial function, eliciting pro-inflammatory responses that can precipitate thrombus formation, underscoring the importance of RRT in evaluating vascular health and disease propensity [68].

Figure 11 reveals that the introduction of an IVC filter corresponds to a reduction in RRT within the affected region. The presence of the filter streamlines blood flow, attenuating regions of stagnation and lessening the propensity for recirculatory zones, leading to a more streamlined and less disrupted flow, hence the observed decrease in RRT. When considering increased clot size, a reduction in RRT proximal to the clot is noted, concurrent with an amplification downstream. The enlarged clot, serving as an augmented obstruction, necessitates an acceleration of blood around it to sustain flow continuity, reducing RRT adjacent to the clot. Conversely, the ensuing flow disturbance downstream is characterized by enhanced recirculation and stagnation, resulting in a slowed blood flow and elevated RRT. The marked pressure gradient imposed by the larger clot further intensifies these dynamics, with flow separation inducing zones of reduced velocity and heightened residence time post-clot. Therefore, consistent with the analyses of OSI and TAWSS, the area downstream of a large clot, exhibiting high RRT, is identified as being at an increased risk for thrombosis.

As illustrated in Figure 12 and Figure 13, the distribution of elevated RRT values indicated by the hyperelastic model, notably in scenarios involving large clots, arises from its ability to account for the elasticity of the vessel wall. Substantial disturbances in blood flow caused by large clots are met with corresponding deformations of the hyperelastic wall, leading to an amplification of flow stagnation zones and an increase in RRT. Such dynamic responses of the wall, in contrast to the rigid wall assumptions, result in observable differences in the predicted locations and magnitudes of RRT maxima. Consequently, when compared to the hyperelastic model, the rigid wall model tends to predict a lower risk of thrombosis, particularly in the regions downstream of large clots.

Based on results shown in Figure 16, similar to observations for OSI and TAWSS, the RRT predictions by laminar and turbulent models generally show little variation due to the predominantly laminar nature of venous flow. However, an exception occurs in the vicinity downstream of a large clot. Here, the flow pattern is substantially disrupted, resulting in increased blood velocity and complex dynamics that may induce turbulence. The turbulent flow model, with its ability to account for vortices and flow separations, predicts a greater RRT in these regions. This heightened sensitivity of RRT, deriving from its relationship with both OSI and TAWSS, makes it more responsive to the transient and fluctuating disturbances brought about by a large clot. Therefore, the turbulent model estimates the RRT to be approximately 10% higher than the laminar model at the location of the most significant disturbance, providing a more nuanced representation of the flow conditions. It is crucial to note, however, that this distinction is significant only in the case of a very large clot within the filter; for other scenarios, the laminar assumption remains a valid approximation.

An analysis of mean RRT values highlights the significant influence of clot size on the differences between models. For small clots, the rigid wall and laminar flow models yield results closely matching those of the hyperelastic wall and turbulent flow models, with differences below 2.5% and 0.4%, respectively. However, in scenarios involving large clots, the rigid wall model underestimates mean RRT by up to 12.06% compared to the hyperelastic model, underscoring its limitations in capturing the increased flow stagnation caused by vessel deformation. Similarly, the turbulent flow model predicts mean RRT values that are up to 10.34% higher than those of the laminar flow model in regions downstream of large clots, where flow disturbances and recirculation zones are more pronounced. These findings suggest that, while the laminar flow and rigid wall models are adequate for smaller clots, their performance declines in accurately predicting mean RRT in the presence of significant flow disruptions caused by large clots.

## 5. Conclusions

This study provides significant insights into the hemodynamic implications of vein wall hyperelasticity and blood flow turbulence in an inferior vena cava (IVC) with an implanted filter. The key findings are summarized as follows:

- The inclusion of hyperelasticity in the vein wall model significantly impacts the distribution and magnitude of hemodynamic parameters, such as TAWSS, OSI, and RRT.
- The presence of a filter and the size of the captured clot notably influence these parameters, with larger clots causing pronounced changes in flow dynamics.
- The rigid wall model, while generally effective, fails to capture the nuanced hemodynamic environment in scenarios with a substantial vessel deformation, particularly in the presence of large clots.
- The laminar flow assumption is valid for cases with empty filters and for small to moderate-sized clots. However, its accuracy diminishes in scenarios involving large clots.
- The turbulence model introduces subtle differences in hemodynamic parameters, which become increasingly evident and clinically significant in cases with larger clots.

These findings underscore the necessity of considering hyperelastic wall properties and turbulence models for accurate hemodynamic simulations, especially under complex conditions involving large clots or substantial vessel deformation.

## 6. Limitations

This study, like all research endeavors, had certain limitations, yet these constraints also provide directions for future work. One of the challenges was the unavailability of patient-specific boundary conditions, leading us to rely on data from previous studies. While this approach ensures consistency with the existing literature, it also opens avenues for future research to incorporate more personalized data, enhancing the relevance and applicability of the findings. Another aspect was the absence of direct clinical data validation. The methodology, initially validated against existing studies due to this limitation, offers a foundational understanding of the subject. However, it also highlights the potential benefits of incorporating clinical data in future studies. Such data could offer more nuanced insights and strengthen the practical applicability of the research.

## Figures and Tables

**Table 1:** Quantitative comparison of the mean hemodynamic parameters predicted by different models.

Clot Size	Index	Hyperelastic	Rigid	% Difference	Turbulent	Laminar	% Difference
Large Clot	TAWSS	4.18	4.433	6.05	4.18	4.13	−1.19
	OSI	0.077	0.074	−4.40	0.077	0.075	−3.24
	RRT	2.292	2.015	−12.06	2.292	2.054	−10.34
Small Clot	TAWSS	0.465	0.479	3.09	0.465	0.464	−0.24
	OSI	0.026	0.025	−1.27	0.026	0.025	−1.85
	RRT	2.314	2.255	−2.55	2.314	2.305	−0.40

## Data Availability

The data used to support the findings of this study are available from the corresponding author upon request.
